# North London or University College Hospital

**Published:** 1904-12-10

**Authors:** Henry Lucas, Walter Baily

**Affiliations:** President; Treasurer; Chairman of Hospital Committee; Vice-Chairman of Hospital Committee


### Editor's Letter-Box.

[Our Correspondents are reminded that prolixity is a great bar to publication, and that brevity of style and conciseness of statement greatly facilitate early insertion.]

### NORTH LONDON OR UNIVERSITY COLLEGE HOSPITAL.

*To the Editor of* The Hospital.

Sir,—The committee again appeal for help to enable them to maintain this hospital and to provide for the sick poor of the district. This help is required more urgently than ever.

The hospital, newly erected through the munificence of the late Sir J. Blundell Maple, is approaching completion. It is expected that 86 additional beds and cots will on January 1st next be ready for occupation, making, with those at present open, a total of 277 beds. This further accommodation will enable the committee to admit many poor patients who cannot now be taken in for want of room. But the increased cost for maintenance will be very great, and is a subject of much anxiety to the committee. It will indeed be a misfortune if the poor cannot reap the full benefit of Sir J. B. Maple's splendid gift owing to sufficient means for its up-keep not being forthcoming. The hospital deals with the enormous number of nearly 50,000 poor patients each year, and is thereby doing an incalculable amount of good.

The committee feel that their case is a good one, and is deserving of serious consideration by the benevolent. They beg most earnestly for largely increased support in the form of annual subscriptions and donations.

Beds or cots may be endowed and named in perpetuity as desired. Payments for the purpose may be made in one sum or by instalments.

Your obedient servants,
Bedford, President.
Monkswell, Treasurer.
Henry Lucas,
Chairman of Hospital Committee.
Walter Baily,
Vice-Chairman of Hospital Committee.

